# Emergency Medicine Perspectives on Quality of Life Outcomes After Emergency Laparotomy: A Systematic Review

**DOI:** 10.7759/cureus.85223

**Published:** 2025-06-02

**Authors:** Amro Abdelaziz Ahmed Mohamed, Hussam Mohamed Ahmed Elawad Elbashir, Yousra Ahmed Abdelrahman Elnasser Ali Elhefnawy, Shaikh Umer Patwa Dania Mohammed, Ashgan Ali Eltayb Abdalla, Alaa Abdelrahman Ahmed Gasmelseed, Mohey Aldien Ahmed Elamin Elnour

**Affiliations:** 1 General Surgery, Naas General Hospital, Naas, IRL; 2 Emergency Medicine and Surgery, King Fahad Hospital, Al Baha, SAU; 3 General Medicine, St. Peter’s Hospital, Surrey, GBR; 4 Medical Claims Operations, Bupa, Jeddah, SAU; 5 Emergency Medicine, Prince Abdul Mohsen Hospital, Al Ula, SAU; 6 Emergency Medicine, Amad Hospital, Riyadh, SAU; 7 Neurosurgery, University Hospital Coventry, Coventry, GBR

**Keywords:** chronic pain, emergency laparotomy, patient-reported outcomes, postoperative recovery, quality of life

## Abstract

Emergency laparotomy is a life-saving intervention for acute abdominal conditions, yet its impact on patients’ long-term quality of life (QOL) remains poorly understood. This systematic review synthesizes evidence on QOL outcomes following emergency laparotomy, with a focus on emergency medicine perspectives, including recovery trajectories, influencing factors, and implications for clinical practice. A comprehensive search of PubMed/Medline, Embase, Web of Science, and Scopus was conducted following the Preferred Reporting Items for Systematic Reviews and Meta-Analyses guidelines. In total, 11 studies were included, encompassing prospective and retrospective cohorts, cross-sectional surveys, and one randomized controlled trial. The risk of bias was assessed using the Newcastle-Ottawa Scale and the Cochrane Risk of Bias tool. Narrative synthesis was performed due to heterogeneity in QOL measures. Key findings revealed significant variability in QOL recovery. Survivors of peritonitis without malignancy reported acceptable QOL, while cancer and advanced age predicted worse outcomes. Chronic pain affected 19-45% of patients, particularly after small bowel obstruction surgery, and was linked to long-term functional impairment. Laparoscopy improved QOL in elderly patients compared to laparotomy. Frailty and prolonged hospitalization were associated with declines in physical and social functioning. Patient-reported outcome measures were feasible in emergency settings but highlighted unmet needs in psychological and social recovery. Emergency laparotomy significantly impacts QOL, with recovery shaped by surgical approach, comorbidities, and postoperative pain. Standardized QOL assessment, integrated multidisciplinary care, and targeted rehabilitation are needed to optimize long-term outcomes. Future research should prioritize prospective studies with uniform QOL metrics to guide patient-centered interventions.

## Introduction and background

Emergency laparotomy is a critical surgical intervention performed for life-threatening abdominal conditions, including perforated viscus, bowel obstruction, peritonitis, and mesenteric ischemia [1]. Despite advances in perioperative care, emergency laparotomy remains associated with significant morbidity, mortality, and long-term functional impairment, raising concerns about its impact on patients’ quality of life (QOL) [2]. While clinical outcomes such as survival rates and postoperative complications have been extensively studied, the long-term QOL following emergency laparotomy has received comparatively less attention, despite its profound implications for patient-centered care and healthcare resource allocation [3]. The existing literature suggests that QOL outcomes are influenced by a complex interplay of factors, including patient demographics, underlying pathology, surgical approach, and postoperative recovery trajectories, yet a comprehensive synthesis of these findings is lacking [4-6].

The emergency medicine perspective emphasizes rapid decision-making, high-acuity settings, and limited preoperative information, which collectively shape the trajectory of patient recovery. These unique features underscore the importance of integrating emergency-specific considerations when evaluating QOL outcomes [5].

The concept of QOL encompasses multidimensional domains, including physical functioning, psychological well-being, social engagement, and symptom burden, each of which may be differentially affected by the physiological stress of emergency surgery and its sequelae [7]. Previous studies have employed diverse methodologies to assess QOL, ranging from generic instruments such as the 36-Item Short Form Health Survey (SF-36) and EuroQol-5 Dimension (EQ-5D) to disease-specific tools such as the Gastrointestinal Quality of Life Index (GIQLI), complicating cross-study comparisons [6]. Furthermore, the emergency medicine perspective on QOL outcomes remains underexplored, particularly regarding triage decisions, postoperative rehabilitation, and the integration of patient-reported outcome measures (PROMs) into acute care pathways [8]. Given the increasing emphasis on value-based healthcare and shared decision-making, understanding the determinants of QOL after emergency laparotomy is essential for optimizing preoperative counseling, tailoring postoperative support, and informing healthcare policy [9].

This systematic review aims to synthesize the available evidence on QOL outcomes following emergency laparotomy, with a particular focus on the emergency medicine perspective. By evaluating studies across diverse healthcare settings and patient populations, we seek to identify consistent patterns, knowledge gaps, and modifiable factors that influence long-term recovery. The review will also critically appraise methodological limitations in the existing literature, including variations in follow-up duration, QOL assessment tools, and risk of bias, to provide recommendations for future research. Ultimately, this work intends to bridge the gap between surgical outcomes and patient-centered care, offering insights that can enhance clinical practice and improve the holistic recovery of patients undergoing emergency laparotomy.

## Review

Methodology

Study Design

This systematic review was conducted following the Preferred Reporting Items for Systematic Reviews and Meta-Analyses (PRISMA) 2020 guidelines to ensure methodological rigor and transparency [10].

Eligibility Criteria

Studies were included if they investigated QOL outcomes in adult patients (≥18 years) following emergency laparotomy, regardless of surgical indication. Only original research articles published in peer-reviewed journals were considered, including randomized controlled trials (RCTs), prospective and retrospective cohort studies, and cross-sectional studies. Case reports, conference abstracts, editorials, and non-English publications were excluded. Studies that did not use validated QOL assessment tools or lacked comparative follow-up data were also excluded.

Information Sources and Search Strategy

A comprehensive literature search was performed across PubMed/Medline, Embase, Web of Science, and Scopus. The search strategy combined Medical Subject Headings (MeSH) terms and free-text keywords related to emergency laparotomy, QOL, patient-reported outcomes, and postoperative recovery. The full search syntax was adapted for each database. Additionally, manual searches of reference lists from included studies and relevant review articles were conducted to identify additional eligible publications.

Study Selection Process

Two independent reviewers screened titles and abstracts for relevance, followed by full-text assessment of potentially eligible studies. Discrepancies were resolved through discussion or consultation with a third reviewer. The selection process was documented in a PRISMA flow diagram, detailing the number of records identified, excluded, and included at each stage, along with reasons for exclusion.

Data Extraction and Management

A standardized data extraction form was developed and pilot-tested to capture key variables systematically. Extracted data included study characteristics such as author, year, country, design, and sample size, as well as patient demographics such as age, sex, and indication for surgery. Details regarding QOL assessment tools, including specific instruments such as SF-36, EQ-5D, and GIQLI, were recorded along with follow-up durations and timepoints. Key QOL findings, such as pre- versus postoperative changes and factors influencing outcomes, were documented. Additionally, any emergency medicine perspectives, including triage decisions or postoperative care considerations, were noted. Data extraction was performed independently by two reviewers, with discrepancies resolved through consensus to ensure accuracy.

Risk of Bias Assessment

The methodological quality of included studies was assessed using the Newcastle-Ottawa Scale (NOS) for non-randomized studies and the Cochrane Risk of Bias Tool (RoB 2.0) [11] for the single RCT. The NOS [12] evaluated selection, comparability, and outcome domains, while RoB 2.0 assessed randomization, deviations, missing data, outcome measurement, and reporting bias. Results were summarized in tables, and studies were categorized as low, moderate, or high risk of bias.

Data Synthesis and Analysis

Due to heterogeneity in study designs, QOL instruments, and follow-up periods, a meta-analysis was deemed inappropriate. Instead, a narrative synthesis was conducted, organizing findings by key themes such as temporal trends in QOL recovery, comparisons of surgical approaches such as laparoscopy versus laparotomy, and factors influencing QOL such as age, comorbidities, and chronic pain. Emergency medicine implications, including the feasibility of PROMs and triage decisions, were also explored.

Results

Search Results

**Figure 1:**
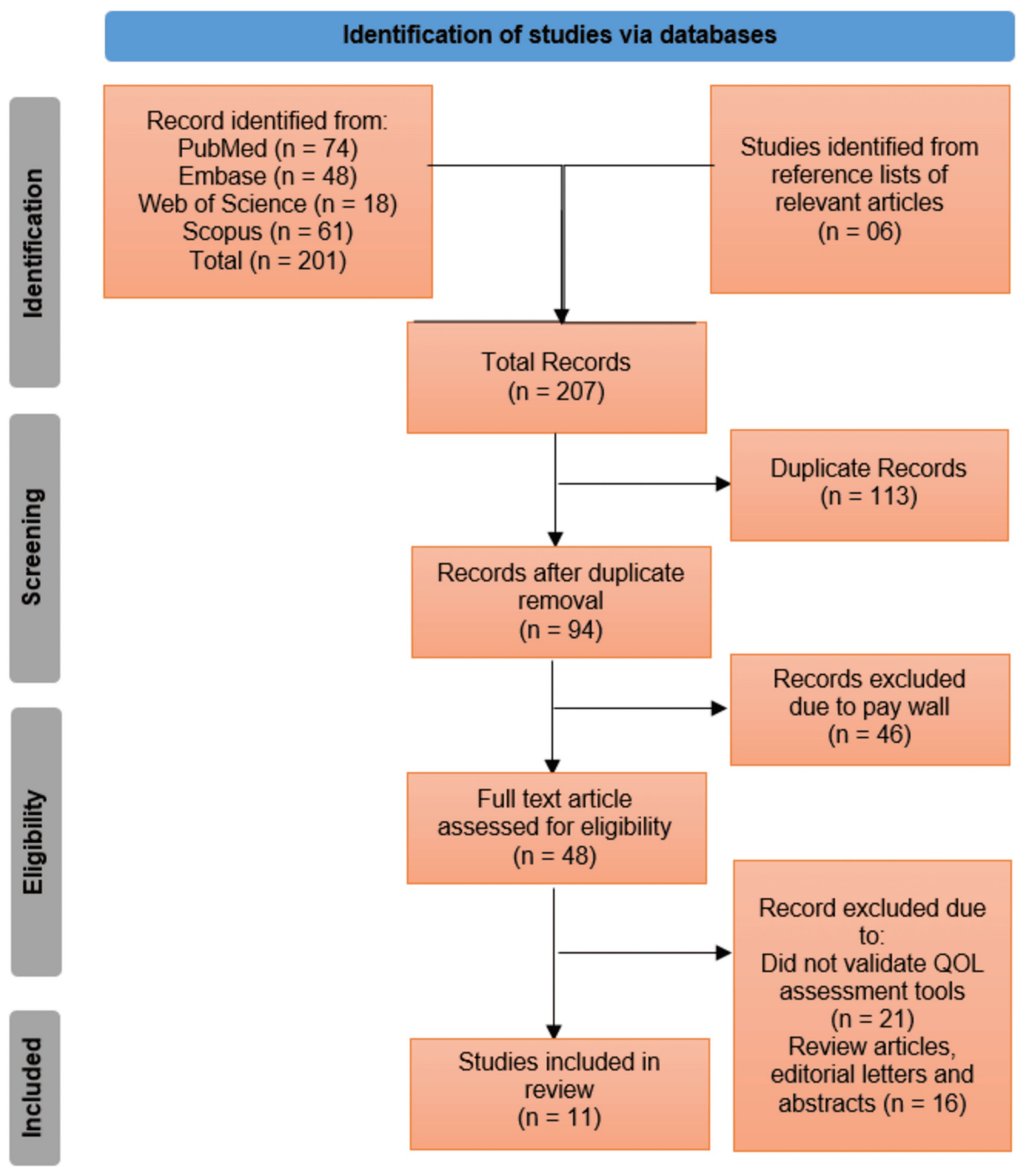
Preferred Reporting Items for Systematic Reviews and Meta-Analyses (PRISMA) 2020 flow diagram detailing the identification and selection of studies.

The systematic search across PubMed (n = 74), Embase (n = 48), Web of Science (n = 18), and Scopus (n = 61) initially identified 201 records, supplemented by six additional studies from reference lists, yielding a total of 207 publications. After removing 113 duplicates, 94 records underwent screening, with 46 excluded due to paywall restrictions. Of the remaining 48 full-text articles assessed for eligibility, 21 were excluded for lacking validated QOL assessment tools, and 16 were reviews, editorials, or abstracts, resulting in 11 studies meeting the inclusion criteria for this review (Figure 1).

Characteristics of Included Studies

This review included 11 studies [13-23] investigating QOL outcomes following emergency laparotomy. The studies were conducted across diverse geographical regions, including Germany [13], India [14], the Netherlands [15], Denmark [16,19], China [17], the United Kingdom [18,20,22], Sub-Saharan Africa [21], and England [20]. Study designs varied, encompassing prospective observational studies [13,15,17,21], retrospective cohort analyses [22,23], cross-sectional surveys [16], and feasibility investigations incorporating PROMs [18,20]. Sample sizes ranged from 51 [14] to 440 patients [19], with populations spanning adults of varying ages, including elderly cohorts [17,22] and patients with specific conditions such as peritonitis [13,15], peptic perforation [14], and acute mesenteric ischemia [23]. Indications for laparotomy were heterogeneous, reflecting the broad scope of emergency surgical interventions. Follow-up durations extended from 1 month [17] to 60 months [19], with QOL assessed using validated tools such as the SF-36 [13], GIQLI [14,16,18,19], EQ-5D [15,18,20,23], and Patient-Reported Outcomes Measurement Information System-25-item (PROMIS-25) [21]. Emergency medicine perspectives were often implicit, focusing on triage, postoperative rehabilitation, or feasibility of PROM collection in acute settings [18,20,22] (Table 1).

**Table 1:** Key characteristics and findings of included studies. QOL: quality of life; SF-36: Short Form-36 Health Survey; GIQLI: Gastrointestinal Quality of Life Index; EQ-5D: EuroQol 5-Dimension; EQ-VAS: EuroQol Visual Analog Scale; HR-QOL: health-related quality of life; AAS: Activity Assessment Scale; S-LANSS: Self-report Leeds Assessment of Neuropathic Symptoms and Signs; RCT: randomized controlled trial; ED: emergency department; PROMs: patient-reported outcome measures; WHODAS 2.0: World Health Organization Disability Assessment Schedule 2.0; POD: postoperative day; PROMIS-25: Patient-Reported Outcomes Measurement Information System-25-item Profile; CPSP: chronic postsurgical pain; NELA: National Emergency Laparotomy Audit; P-POSSUM: Portsmouth-Physiological and Operative Severity Score for the Enumeration of Mortality and Morbidity; AMI: acute mesenteric ischemia; NHS: National Health Service; IQR: interquartile range

Study (author, year)	Country	Study design	Sample size	Population details	Indication for laparotomy	QOL measurement tool	Follow-up duration	Key findings on QOL	Emergency medicine perspective (e.g., ED admission, timing, triage)
Scheingraber et al. [13] 2002	Germany	Prospective observational study	136	Patients with severe peritonitis admitted between January 1996 to May 1999	Severe peritonitis	SF-36	Up to 1 year	Acceptable QOL in survivors without malignancy; cancer patients had worse physical/emotional outcomes; age reduced physical function	The study implies the critical care context involving emergency admission
Joneja et al. [14] 2004	India	Prospective study	51	Adult survivors of peptic perforation undergoing surgery	Peptic perforation	GIQLI	Preoperative, 3 months, 6 months	Significant improvement in GIQLI scores across all domains at 3 and 6 months; QOL near normal by 6 months; no age/gender difference	Patients presented acutely for emergency surgical intervention
Boer et al. [15] 2007	The Netherlands	Prospective cohort	130	Patients with secondary peritonitis	Secondary peritonitis	EQ-5D and EQ-VAS	6 months	HR-QOL significantly worse than healthy population; longer hospital stay and enterostomy negatively affected QOL	Initial disease severity not predictive; disease course (e.g., hospital stay) had more impact
Jeppesen et al. [16] 2016	Denmark	Questionnaire-based, cross-sectional study	90	Patients post-emergency laparotomy for small bowel obstruction	Small bowel obstruction	GIQLI, AAS, S-LANSS	Not explicitly stated (follow-up implied)	Patients with chronic pain had significantly lower GIQLI scores (109 vs. 127, p < 0.001)	Not specifically discussed
Li et al. [17] 2017	China	RCT	100 (50 laparoscopy, 50 laparotomy)	Elderly patients (>60 years) with upper GI ulcer perforation	Upper gastrointestinal ulcer perforation	QOL scores	1 month and 3 months postoperatively	The laparoscopy group had significantly improved QOL at both follow-ups compared to the laparotomy group	All patients received emergency surgery, reflecting ED-level urgency
Kwong et al. [18] 2018	UK	Observational study with retrospective and prospective elements	255 contacted; 190 responded	Patients undergoing emergency laparotomy for GI conditions in 11 hospitals	Gastrointestinal conditions	EQ-5D-3L and GIQLI	3 months (mailed follow-up)	Significant improvement in GIQLI total and symptom subscale; no change in emotional/physical aspects or EQ-5D overall; deterioration in social subscale	PROMs collected during admission and after discharge, assessing the feasibility of outcome measurement in the emergency surgery context
Tolstrup et al. [19] 2019	Denmark (Copenhagen University Hospital Herlev)	Questionnaire-based cohort study	440 (out of 605 eligible, from 1573 initial population)	Adults, median age 69 (range 18–95), 56.4% female, patients undergoing emergency midline laparotomy	Not specified (emergency laparotomy cases in general)	GIQLI; Activity Assessment Scale	Median 60 months (IQR = 47)	19% had CPSP with low GI QoL; 45% had moderate–severe functional impairment	Emergency midline laparotomy setting; likely ED admissions, but not explicitly detailed in abstract
Saunders et al. [20] 2021	England	Feasibility longitudinal observational study	70 (out of 129 eligible)	Consecutive patients undergoing emergency laparotomy at a single NHS center	Not reported	EQ-5D and WHODAS 2.0	1, 3, 6, and 12 months	Demonstrated feasibility of collecting PROMs; first longitudinal data on QOL up to 1 year post-surgery	Patients identified from the local NELA register; reflects the integration of emergency data in recruitment
Purcell et al. [21] 2021	Sub-Saharan Africa	Prospective observational	117	Adult patients, median age 39 years (IQR 27–54), 76.1% male	Intestinal volvulus (28.3%), intestinal perforation (27.4%)	PROMIS-25, Index of Independence in Activities of Daily Living	Pre-op, POD #1, POD #7, POD #30	High pre-op anxiety, depression, fatigue, pain; improvement postoperatively. Mobility was poor, and pain intensity improved.	HRQOL poor preoperatively, improved postoperatively. Pain control and mobility remain key concerns for postoperative recovery.
Alder et al. [22] 2021	UK	Retrospective cohort	153	Septuagenarian population (median age 79 years, 1.7 men to women)	Acute intra-abdominal pathology	Subjective postoperative functioning assessment	1 to 2 years	Decline in physical functioning, lower energy, higher fatigue, reduction in social functioning, increase in clinical frailty scale score	Risk stratification with mortality scoring (P-POSSUM, NELA-adjusted risk) and frailty assessment, emphasis on postoperative rehabilitation
Witte et al. [23] 2022	Germany (University Medical Center Rostock)	Retrospective analysis	64	Patients with occlusive arterial or venous mesenteric ischemia treated operatively between 2008 and 2016	AMI	EQ-5D	Long-term (not specified)	Long-term survivors had impaired QOL compared to the reference population. Impairment due to comorbidities, not disease-specific sequelae	Focus on comorbidities and operative outcome, no stoma patients, analysis of leak rates in anastomosis

Quality of Life Outcomes Following Emergency Laparotomy

Preoperative and postoperative QOL trajectories revealed significant variability across studies. Survivors of severe peritonitis without malignancy reported acceptable QOL, though physical function declined with age, and cancer diagnoses worsened outcomes [13]. Patients with peptic perforation demonstrated near-normal QOL by six months post-surgery, with no age or gender disparities [14]. In contrast, secondary peritonitis patients exhibited persistently poorer QOL at six months compared to healthy populations, with prolonged hospitalization and enterostomy further impairing mobility and self-care [15]. Chronic postoperative pain was a critical determinant of reduced QOL, particularly after small bowel obstruction surgery, where patients with pain scored significantly lower on GIQLI (109 vs. 127, p < 0.001) [16]. Minimally invasive approaches, such as laparoscopy, improved QOL in elderly patients with upper gastrointestinal perforations, underscoring the benefits of reduced surgical trauma [17].

Longitudinal assessments highlighted the feasibility of PROM collection in emergency settings, though social functioning often deteriorated despite improvements in physical symptoms [18]. Chronic postsurgical pain (CPSP) and functional impairment affected 19% and 45% of patients, respectively, after emergency midline laparotomy, emphasizing the need for long-term pain management strategies [19]. Studies employing serial follow-ups (e.g., at 1, 3, 6, and 12 months) noted gradual QOL recovery, though pre-injury baselines were rarely fully restored [20]. In resource-limited settings, preoperative anxiety, depression, and pain were prevalent, with mobility remaining a persistent challenge postoperatively [21]. Frailty and advanced age were strongly associated with declining physical and social functioning, reinforcing the importance of preoperative risk stratification [22]. For long-term survivors of acute mesenteric ischemia, preexisting comorbidities, rather than disease-specific sequelae, drove QOL impairments [23] (Table 2).

**Table 2:** Summary of QOL outcomes following emergency laparotomy. QOL: quality of life; SF-36: Short Form-36 Health Survey; GIQLI: Gastrointestinal Quality of Life Index; EQ-5D: EuroQol 5-Dimension; EQ-VAS: EuroQol Visual Analog Scale; HR-QOL: health-related quality of life; AAS: Activity Assessment Scale; S-LANSS: Self-report Leeds Assessment of Neuropathic Symptoms and Signs; RCT: randomized controlled trial; ED: emergency department; PROMs: patient-reported outcome measures; WHODAS 2.0: World Health Organization Disability Assessment Schedule 2.0; POD: postoperative day; PROMIS-25: Patient-Reported Outcomes Measurement Information System-25-item Profile; CPSP: chronic postsurgical pain; NELA: National Emergency Laparotomy Audit; P-POSSUM: Portsmouth Physiological and Operative Severity Score for the Enumeration of Mortality and Morbidity; AMI: acute mesenteric ischemia; NHS: National Health Service; IQR: interquartile range; VAS: Visual Analog Scale; APSP: acute postsurgical pain; CCI: Charlson Comorbidity Index

Study (author, tear)	QOL tool used	Preoperative QOL reported?	Domains assessed	Timepoints of assessment	Change in QOL over time	Factors affecting QOL	Clinical implications
Scheingraber et al. [13] 2002	SF-36	No	Physical function, emotional function	Within the first year post-discharge	Physical functions were reduced; emotional impairment noted later in cancer patients	Age (↓ physical function), Cancer (↓ physical + emotional)	Survivors without malignancy had acceptable QOL compared to general population. QOL assessments support treatment intensity, but not predictive at the individual level
Joneja et al. [14] 2004	GIQLI	Yes	GI core, GI disease-specific, psychological, physical, and social components	Preoperative, 3 months, 6 months	Significant improvement in overall QOL and all domains at both 3 and 6 months (p < 0.001); QOL approached near-normal by 6 months	No significant variation by age or gender	Peptic perforation does not lead to long-term QOL impairment; patients show significant recovery in QOL by 6 months post-surgery
Boer et al. [15] 2007	EQ-5D, EQ-VAS	No	Mobility, self-care, usual activities, pain/discomfort, anxiety/depression (EQ-5D); overall health status (EQ-VAS)	6 months post-operatively	QOL significantly worse in all domains compared to a healthy population at 6 months. No improvement data over time reported	Longer hospital stays and presence of enterostomy negatively impacted mobility, self-care, and daily activities. Doubling of hospital stay decreased EQ-VAS by 3.8 points (p = 0.015)	Early disease severity was not predictive of poor QOL, but complicated postoperative course and prolonged hospitalisation were key predictors of poorer QOL outcomes at 6 months
Jeppesen et al. [16] 2016	GIQLI, AAS, S-LANSS	No	Gastrointestinal function, pain, functional impairment, presence of incisional hernia	Single follow-up post-operatively (specific timepoint not provided)	Patients with chronic postoperative pain had significantly lower GIQLI scores compared to those without (109 vs. 127)	Chronic postoperative pain, pain-related functional impairment, incisional hernia	Chronic pain after emergency laparotomy is common and linked to reduced QOL; highlights the need for long-term follow-up and potential interventions; findings should be confirmed in prospective studies
Li et al. [17] 2017	QOL scoring tool	No	General quality of life (unspecified domains); postoperative pain (VAS); Functional recovery	1 month and 3 months postoperatively	Improved QOL scores in laparoscopy group compared to laparotomy at both 1 and 3 months	Surgical approach (laparoscopy vs. laparotomy); postoperative pain; Inflammatory cytokine levels (hs-CRP, TNF-α, IL-6)	Laparoscopy improves QOL outcomes in elderly patients by reducing pain, complications, and inflammatory burden; supports minimally invasive approach in appropriate emergency cases
Kwong et al. [18] 2018	EQ-5D-3L, GIQLI	Yes (retrospective)	GIQLI: Symptoms, emotion, physical, social EQ-5D-3L: Overall health status	Baseline (retrospective at admission), 3 months postoperatively	GIQLI: Improvement in overall and symptom subscale (93.3 → 97.9, p = 0.048; 51.9 → 59.6, p < 0.001). EQ-5D-3L: Non-significant improvement (0.58 → 0.64, p = 0.06). GIQLI Social: Decline (11.0 → 9.8, p = 0.0006)	Older, female, more affluent patients more likely to respond (response bias minimal)	Demonstrates feasibility of using PROMs post-emergency laparotomy; highlights importance of including social recovery in postoperative care and recognizing patient perspectives in surgical outcomes
Tolstrup et al. [19] 2019	GIQLI; Activity Assessment Scale	No	Gastrointestinal quality of life; pain-related functional impairment	Median follow-up: 60 months (IQR = 47)	19% had CPSP and low GI-QOL; 45% reported moderate–severe functional impairment	APSP (OR = 5.0); Age < 60 (OR = 2.1)	High burden of CPSP and functional impairment highlights the need for long-term follow-up and pain management strategies in emergency laparotomy patients
Saunders et al. [20] 2021	EQ-5D, WHODAS 2.0	Yes	Mobility, self-care, usual activities, pain/discomfort, anxiety/depression (EQ-5D); functioning and disability (WHODAS)	Baseline, 1, 3, 6, and 12 months postoperatively	Longitudinal data demonstrated a trajectory of recovery across all domains, with QOL gradually improving over 12 months but not necessarily returning to baseline	Not explicitly detailed in abstract	Demonstrates feasibility of longitudinal PROM collection post- emergency laparotomy; supports the need for integrating QOL outcomes into routine postoperative evaluation and study design
Purcell et al. [21] 2021	PROMIS-25 and Index of Independence in Activities of Daily Living	Yes	Anxiety, depression, fatigue, pain interference, pain intensity, mobility	Preoperative (up to POD#1), POD#7, POD#30	Anxiety, depression, fatigue, and pain interference were high preoperatively but improved by POD#30; mobility remained poor throughout; pain intensity reduced from 10/10 preoperative to 3/10 at POD#30	Presence of postoperative complications was associated with significantly worse HRQOL in all domains by POD#30	Highlights poor preoperative HRQOL in resource-limited settings; Emphasizes need for interventions to improve mobility and pain management during recovery
Alder et al. [22] 2021	Not specified (subjective follow-up)	Partially (preoperative frailty score and risk via P-POSSUM mentioned)	Physical functioning, energy, fatigue, social functioning, frailty	Median follow-up of 19 months (1–2 years postoperatively)	Decline in physical functioning, lower energy, higher fatigue, reduced social functioning; increase in frailty	Preoperative Clinical Frailty Scale	QOL significantly declines post-emergency laparotomy in older adults; frailty should guide preoperative counselling. Emphasis needed on rehabilitation, holistic recovery, and realistic expectation-setting
Witte et al. [23] 2022	EQ-5D	Yes	Five dimensions: mobility, self-care, usual activities, pain/discomfort, anxiety/depression	Long-term (after discharge, exact timepoints not specified)	Significantly impaired compared to age- and sex-matched reference population	Higher CCI	Long-term survivors of AMI have impaired QOL, not due to disease-specific sequelae but due to preexisting comorbidities

Key Factors Influencing QOL Outcomes

Several factors consistently influenced QOL outcomes across studies. Age and malignancy were linked to poorer physical and emotional functioning [13,22], while surgical approach (laparoscopy vs. laparotomy) significantly impacted recovery trajectories in elderly populations [17]. Prolonged hospital stays and enterostomy were associated with worse QOL in peritonitis patients [15], and chronic pain emerged as a major contributor to long-term disability [16,19]. Social and emotional domains were often overlooked, yet declines in these areas were notable in studies assessing multidimensional QOL [18,20]. Frailty, as measured by clinical scoring systems, provided a robust predictor of postoperative QOL decline, particularly in older adults [22]. Collectively, these findings underscore the multifactorial nature of QOL recovery, necessitating tailored interventions addressing physical, psychological, and social dimensions.

Quality Assessment Results

The risk of bias assessment using the NOS for non-randomized studies revealed that two studies demonstrated low risk of bias [15,19], scoring 8/9 due to robust selection criteria and comparability adjustments, while Witte et al. [23] and Scheingraber et al. [13] also achieved low risk with scores of 7/9. The majority of studies [14,16,18,20-22] were rated as moderate risk, primarily due to limitations in comparability or outcome assessment (Table 3).

**Table 3:** Risk of bias using the Newcastle-Ottawa Scale tool.

Study (author, year)	Study design	Selection (maximum = 4)	Comparability (maximum = 2)	Outcome (maximum = 3)	Total score (maximum = 9)	Risk of bias
Scheingraber et al. [13] 2002	Prospective observational	3	2	2	7	Low
Joneja et al. [14] 2004	Prospective study	3	1	2	6	Moderate
Boer et al. [15] 2007	Prospective cohort	4	2	2	8	Low
Jeppesen et al. [16] 2016	Cross-sectional	3	1	2	6	Moderate
Kwong et al. [18] 2018	Observational (retrospective and prospective)	3	1	2	6	Moderate
Tolstrup et al. [19] 2019	Cohort study	4	2	2	8	Low
Saunders et al. [20] 2021	Longitudinal observational	3	1	2	6	Moderate
Purcell et al. [21] 2021	Prospective observational	3	1	2	6	Moderate
Alder et al. [22] 2021	Retrospective cohort	3	1	2	6	Moderate
Witte et al. [23] 2022	Retrospective analysis	3	1	3	7	Low

For the single RCT [17], the RoB 2.0 tool indicated low overall risk, with no critical flaws in randomization, outcome measurement, or reporting, though the lack of blinding posed some concerns (Table 4).

**Table 4:** Risk of bias using the Cochrane Risk of Bias 2.0 tool.

Study	Study design	Randomization process	Deviations from intended interventions	Missing outcome data	Measurement of outcome	Selection of reported result	Overall risk of bias
Li et al. [17] 2017	Randomized controlled trial	Low risk	Low risk	Low risk	Low risk	Low risk	Low risk

Discussion

This study provides a comprehensive synthesis of QOL outcomes following emergency laparotomy, highlighting both the immediate and long-term impacts of this life-saving surgical intervention. Across the 11 included studies, several consistent themes emerged regarding the trajectory of QOL recovery, the factors influencing outcomes, and the implications for emergency medicine practice. The review underscores that while emergency laparotomy is often successful in addressing acute abdominal pathologies, its aftermath can significantly alter patients’ physical, emotional, and social well-being, with recovery patterns varying widely depending on patient-specific and surgical factors.

One of the most striking observations from this review is the heterogeneity in QOL outcomes, which reflects the diverse indications for emergency laparotomy and the varying baseline health statuses of patients. For instance, survivors of severe peritonitis without malignancy reported acceptable QOL, though physical function declined with age, and cancer diagnoses exacerbated emotional and physical impairments [13]. This aligns with broader literature on post-surgical recovery, where malignancy and advanced age are consistently associated with poorer functional outcomes [24]. Similarly, patients with peptic perforation demonstrated near-normal QOL by six months post-surgery, suggesting that certain conditions may allow for more complete recovery [14]. This finding contrasts with studies on secondary peritonitis, where QOL remained significantly worse than healthy populations at six months, driven by prolonged hospital stays and the need for enterostomy [15,19]. Such disparities highlight the importance of considering the underlying pathology when counseling patients about expected recovery trajectories.

Chronic pain emerged as a critical determinant of long-term QOL impairment, particularly in studies focusing on small bowel obstruction and midline laparotomy [16]. The prevalence of CPSP in these populations (19-45%) mirrors findings in other surgical cohorts, where persistent pain is linked to functional disability and reduced QOL [25]. The high burden of CPSP underscores the need for standardized pain management protocols and long-term follow-up, as current practices often prioritize acute postoperative care over chronic symptom management. This gap is particularly evident in resource-limited settings, where preoperative [19] depression and pain are prevalent, and mobility remains a persistent challenge postoperatively [21]. These findings echo global concerns about the under-recognition and undertreatment of chronic pain, particularly in low- and middle-income countries [26].

The review also highlights the potential benefits of minimally invasive approaches, such as laparoscopy, in improving QOL outcomes. Li et al. [17] demonstrated that elderly patients undergoing laparoscopic repair for upper gastrointestinal perforation had significantly better QOL at one and three months compared to those undergoing laparotomy. This aligns with a growing body of evidence supporting laparoscopy in emergency settings, where reduced surgical trauma translates to faster recovery and fewer complications [27]. However, the applicability of these findings may be limited by patient factors (e.g., hemodynamic instability) and resource constraints, particularly in settings where laparoscopic expertise is scarce. Future research should explore barriers to adopting minimally invasive techniques in emergency laparotomy and their cost-effectiveness in diverse healthcare systems.

The integration of PROMs into emergency laparotomy care pathways was another key theme. Studies by Kwong et al. [18] and Saunders et al. [20] demonstrated the feasibility of collecting PROMs in acute settings, though they also revealed challenges, such as declining social functioning despite improvements in physical symptoms. These findings resonate with broader calls to incorporate PROMs into routine postoperative care, as they provide valuable insights into patient-centered outcomes that are often overlooked in traditional clinical metrics [28]. However, the variability in QOL assessment tools across studies, ranging from generic (e.g., SF-36, EQ-5D) to disease-specific (e.g., GIQLI), complicates cross-study comparisons and underscores the need for standardized measurement protocols. This limitation is not unique to emergency laparotomy; it reflects a broader issue in surgical outcomes research, where the lack of consensus on QOL tools hinders data synthesis and meta-analyses [29].

Frailty and advanced age were consistently associated with poorer QOL outcomes, particularly in studies focusing on elderly populations [22]. Alder et al. [22] reported significant declines in physical and social functioning among septuagenarians, with frailty scores predicting worse outcomes. These findings align with existing literature on geriatric surgery, where frailty is increasingly recognized as a critical determinant of postoperative recovery [30]. The integration of frailty assessments into preoperative workflows, as suggested by these studies, could enhance risk stratification and inform shared decision-making. However, the generalizability of these findings may be limited by the predominance of high-income country data, where access to geriatric-specific perioperative care is more readily available. Future studies should explore frailty’s impact in resource-limited settings, where comorbidities and delayed presentations may exacerbate postoperative decline.

The emergency medicine perspective on QOL outcomes remains underexplored in the included studies, though implicit themes emerged regarding triage decisions, postoperative rehabilitation, and the feasibility of outcome measurement in acute settings. For example, Kwong et al. [18] and Saunders et al. [20] emphasized the logistical challenges of PROM collection during and after emergency admissions, while Purcell et al. [21] highlighted the high prevalence of preoperative psychological distress in resource-limited settings. These findings suggest that emergency laparotomy pathways should incorporate multidisciplinary support, including mental health services and rehabilitation planning, to address the holistic needs of patients. This approach is supported by broader literature on emergency surgery, where integrated care models have been shown to improve both clinical and QOL outcomes [31].

Comparisons with existing studies not included in this review further contextualize these findings. For instance, the observed decline in social functioning post-laparotomy mirrors findings in elective abdominal surgery cohorts, where postoperative social isolation and reduced participation in activities are common [32]. Similarly, the association between prolonged hospital stays and poorer QOL aligns with research on critical care survivors, where intensive care unit length of stay is a known predictor of long-term functional impairment [33]. However, the unique physiological and psychological stressors of emergency surgery, such as unplanned hospitalization, acute pain, and existential distress, may exacerbate these effects, underscoring the need for tailored interventions.

Study limitations

This review has several limitations. First, the heterogeneity in study designs, QOL assessment tools, and follow-up durations precluded a meta-analysis, limiting the ability to quantify pooled effects. Second, the predominance of observational studies introduces risks of bias, particularly in confounding and selection bias, though the use of validated quality assessment tools mitigates this concern. Third, the under-representation of low- and middle-income country data restricts the generalizability of findings to resource-limited settings, where surgical outcomes may differ due to variations in healthcare infrastructure and patient demographics. Finally, the focus on QOL outcomes may overlook other important endpoints, such as cost-effectiveness or caregiver burden, which are critical for holistic healthcare planning.

## Conclusions

While emergency laparotomy effectively addresses life-threatening abdominal conditions, its sequelae, particularly chronic pain, functional decline in frail and elderly populations, and the psychosocial toll of prolonged hospitalization, frequently undermine postoperative well-being. Notably, minimally invasive approaches such as laparoscopy show promise in improving recovery trajectories, while the feasibility of PROMs in emergency settings underscores the value of integrating patient perspectives into care pathways. There is an urgent need to reorient emergency surgical care beyond survival metrics toward holistic recovery. The heterogeneity in QOL assessment tools across studies highlights a methodological gap that must be addressed through standardized protocols, while the paucity of data from resource-limited settings calls for globally inclusive research. For clinicians, these findings mandate a paradigm shift: preoperative frailty assessments, tailored rehabilitation programs, and multidisciplinary pain management strategies should become integral components of emergency laparotomy pathways. Future research must prioritize prospective, multicenter studies to elucidate modifiable predictors of QOL and evaluate targeted interventions; only then can we ensure that life-saving surgery does not come at the cost of long-term patient well-being.
